# Surface warming from altitudinal and latitudinal amplification over Antarctica since the International Geophysical Year

**DOI:** 10.1038/s41598-023-35521-w

**Published:** 2023-06-12

**Authors:** Aihong Xie, Jiangping Zhu, Xiang Qin, Shimeng Wang, Bing Xu, Yicheng Wang

**Affiliations:** 1 State Key Laboratory of Cryospheric Sciences, Northwest Institute of Eco-Environment and Resources, Chinese Academy of Sciences, Lanzhou, China; 2 University of Chinese Academy of Sciences, Beijing, China; 3 Weather Modification Office of Liaoning Province, Shenyang, China; 4 Lanzhou Central Meteorological Observatory, Lanzhou, China

**Keywords:** Climate change, Cryospheric science

## Abstract

Warming has been and is being enhanced at high latitudes or high elevations, whereas the quantitative estimation for warming from altitude and latitude effects has not been systematically investigated over Antarctic Ice Sheet, which covers more than 27 degrees of latitude and 4000 m altitude ranges. Based on the monthly surface air temperature data (1958–2020) from ERA5 reanalysis, this work aims to explore whether elevation-dependent warming (EDW) and latitude-dependent warming (LDW) exist. Results show that both EDW and LDW have the cooperative effect on Antarctic warming, and the magnitude of EDW is stronger than LDW. The negative EDW appears between 250 m and 2500 m except winter, and is strongest in autumn. The negative LDW occurs between 83 °S and 90 °S except in summer. Moreover, the surface downward long-wave radiation that related to the specific humidity, total cloud cover and cloud base height is a major contributor to the EDW over Antarctica. Further research on EDW and LDW should be anticipated to explore the future Antarctic amplification under different emission scenarios.

## Introduction

Regions with high elevation often exhibit a stronger warming signal relative to the same latitude area, and this phenomenon is referred as elevation-dependent warming (EDW), which is a notable characteristics under global warming^1–4^. EDW has been explored in many places, such as Tibetan Plateau (TP)^3,5,6^, Eastern Siberian Arctic^7^, Eastern Alps^8^, Hawaiian Islands^9^, Kilimanjaro^10^, Himalayas^11^, and Chinese Tian Shan^12^, etc. Some studies have reported the negative EDW, which defines the warming reduction at the highest elevations, and the EDW may illustrate different features in different sub-regions and timescales^3^. However, more and more studies have found that the warming amplification has been strengthened with elevation^13–16^, and the EDW strength is affected by many factors including but not limited to the mountain magnitude, the height, the topography and the climatic regime^17^. For example, the rapid annual warming during the period 1961–2007 can be observed in the relatively low elevations over the northern TP, indicating the occurrence of negative EDW and the regional difference of EDW^18^. In contrast, the clear EDW has been found over the whole TP during 1961–1990, especially above 2000 m^19^.

Latitude-dependent Warming (LDW) has also received a lot of attention in recent years. The warming rates of near-surface temperature enhance as the latitude increases over the Arctic, and have been demonstrated as Arctic Amplification (AA) in recent decades based on observational data, reanalyses and climate model simulations^20–22^. AA is almost four times the global means^23^, and has accelerated during recent decades especially in the last 10 years, and the warming has been most significant in the cold season months (autumn and winter). AA occurs in the whole troposphere, which has a wide influence, and is related to the local feedback and poleward heat and moisture transport from lower latitudes^24^. However, little attempt has been undertaken to quantify the relationship of the changes in temperature with both altitude and latitude in Antarctica.

**Figure 1:**
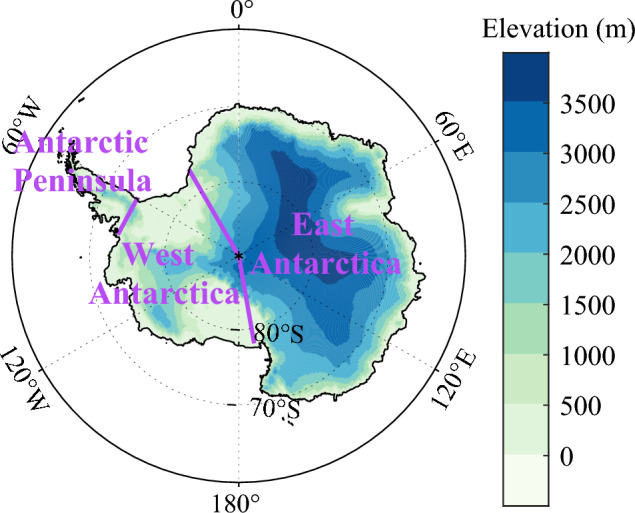
The topographic map of Antarctic Ice Sheet. The purple panel shows the boundaries of the three Antarctic sub-region of East Antarctica, West Antarctica and Antarctic Peninsula. The map was drawn in the software MATLAB R2018b (https://ww2.mathworks.cn/products/matlab.html).

Antarctica, the largest huge ice sheet, is an important contributor to the rise in global sea-levels^25,26^. Moreover, polar amplification is an established scientific fact with the global warming, although the amplification is weak over Antarctica, which may relate to the ocean heat uptake and deep mixing in the Southern Ocean^21,27–29^. The elevation of Antarctic Ice Sheet (AIS) fluctuates greatly, with the highest altitude up to 4892 m at Mount Vinson, while the lowest below sea level^30^ (Fig. 1). The temperature changes over AIS is inhomogeneous, and Antarctic Peninsula (AP) has experienced a strong warming in the second half of the twentieth century^31–33^. However, the warming signal may be reversed in short time periods, such as the cooling since the late 1990s^34,35^, and the cooling is most significant in the northern and north-eastern side of the AP and the South Shetland Islands^36^, and the warming pause may end in the mid-2010s, which is related to the changes in the large-scale climate modes^37^. Most notably, the warming in AP always can be observed in periods long than 30 years, and cooling in recent years is not the evidence of the shift in the overall warming trend^38^. The strong warming also occurs in West Antarctic Ice Sheet (WAIS), which results in surface melting over the WAIS and contributes to sea level rise^39–41^. Differently, the temperature change over East Antarctic Ice Sheet (EAIS) is enigmatic. Most studies reveal that the general cooling is the main trend after 1950s^35,42–44^, while the warming tendency can be captured in austral spring during the period 1979–2019^4^. Many studies have explored the mechanism of Antarctic temperature changes from many perspectives such as the Amundsen Sea low (ASL), Southern Annular Mode (SAM)^45,46^, La Niña events^44,47^, sea surface temperature^48^, sea ice^49^, westerlies, regional atmospheric circulation^50^ and Antarctic ozone hole^51^. However, no quantification has yet been separated for the surface warming from altitudinal and latitudinal amplification over Antarctica.

In this study, we firstly analyze the Antarctic temperature changes on annual and seasonal scales. Based on the elevation band and latitude band methods, then we explore to what extent Antarctic temperature trends can be explained by altitude and latitude effect.

## Data and methods

### Data sources

The observational data is scarce in Antarctica, and most weather stations are located at the coastal areas^52,53^. Therefore, reanalysis data is preferred and widely used in Antarctica. Compared with the other reanalysis, the Fifth Generation Global Atmospheric reanalysis data (ERA5) developed by European Centre for Medium-Range Weather Forecasts (ECMWF) has the better performance on representing Antarctic temperature, which has high correlation coefficients and low bias compared to the measured data, and the high skill in representing the temperature over Antarctica contributes to the improvements in observation operators, model physics, core dynamics and data assimilation^53,54^. ERA5 provides temperature reanalysis data since 1950. Although ERA5 reproduce quite well the Antarctic temperature for the period 1950–1979^53,55^, there is a drop in the performance at AP previous to 1957^56^. In this study, we employ ERA5 reanalysis, and select the 0.25° × 0.25° gridded dataset of monthly surface air temperature from 1958 to 2020, downloaded from ECMWF https://cds.climate.copernicus.eu/#!/search?text=ERA5&type=dataset.

We calculate the variation trend through linear regression according to the anomalies (relative to 1961–1990 mean), and F test is used to estimate the significance (*p* < 0.05) of the trends. The regional trend has been computed from the average anomalies of the all grids across the AIS.

### Detection of Antarctic amplification

Similar to Arctic amplification (AA)^57^, the Antarctic amplification (AnA) can be defined as the ratio of the value of the linear trend value of average surface air temperature over the grids in AIS to the trend of average temperature for whole grids in Southern Hemisphere (SH). Most of the SH is covered by ocean, and therefore have a lower temperature trend. To eliminate the influence of ocean, this study also analyze the AnA based on the ratio of the trend over AIS and that only for land region of SH.

### Detection of altitudinal and latitudinal amplification trends

In the process of exploring the EDW and LDW, the band method is employed to explore the altitudinal and latitudinal amplification, which is widely used to investigate EDW^58,59^. The detailed method is as the following: For the elevation band method, we divide into 250 m wide elevation bands starting at − 250 m, calculate the linear trend for each band based on the temperature anomalies, and regress the band trends against band mean altitudes. Then the slope is the altitudinal amplification trend. Similarly, the latitude has been grouped into 1.0° wide bands starting at 63 °S, and the latitudinal amplification trend is calculated by regressing the band trends against the mean latitude band.

Note: The EDW and LDW in 1958–2020 is basically consistent with the results in 1979–2020, and the accuracy of ERA5 data in AIS is reliable after 1979, which indicates that the corresponding results during the period 1958–2020 are confident.

## Results

### Antarctic amplification assessment during 1958–2020

**Figure 2:**
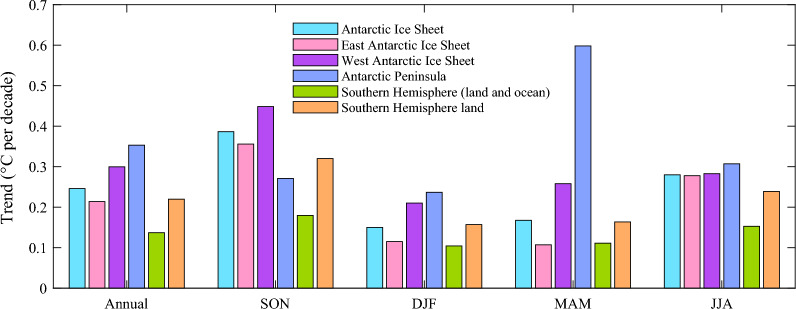
Regionally average annual and seasonal (spring, SON; summer, DJF; autumn, MAM; winter, JJA) mean surface temperature trends for 1958–2020 over the Antarctic Ice Sheet, East Antarctic Ice Sheet, West Antarctic Ice Sheet, Antarctic Peninsula, and Southern Hemisphere. All trends are significant above the 95% confidence interval.

Compared to the near-surface temperature change of SH, AIS display stronger warming tendency (Fig. 2), which indicates the occurrence of AnA. For AIS, warming signal is most conspicuous in austral spring (September–November, SON), with the trend of 0.39 °C per decade, and weakest warming can be observed in austral summer (December–February, DJF). In addition, the variations in temperature over AIS exhibit regional differences. On annual scale, the greatest warming occurs in AP, followed by WAIS, and weakest in EAIS. The AnA appears when compared to the changes in whole SH (Table 1), and the amplification index for annual, spring, summer, autumn and winter are 1.80, 2.15, 1.44, 1.51 and 1.83, respectively. WAIS and AP display annual and seasonal warming amplification, and the amplification in EAIS is absent in austral autumn (March–May, MAM). When compared with the tendency only for land region, the intensify of AnA weakens, and even disappears in austral summer. The amplification of EAIS only appears in spring and winter (June–August, JJA), and AP fails to capture the amplification signal in spring. Generally, although the regional differences exist, a slight AnA can be observed during the period 1958–2020.

**Table 1:** Annual and seasonal (spring, SON; summer, DJF; autumn, MAM; winter, JJA) Antarctic amplification index over all Antarctic Ice sheet (AIS) and its sub-regions of East Antarctic Ice sheet (EAIS), West Antarctic Ice sheet WAIS) and Antarctic Peninsula (AP).

	Annual	SON	DJF	MAM	JJA
Compared to Southern Hemisphere (land and ocean)					
AIS	1.80	2.15	1.44	1.51	1.83
EAIS	1.56	1.98	1.10	0.97	1.82
WAIS	2.19	2.49	2.02	2.32	1.85
AP	2.58	1.51	2.27	5.38	2.01
Compared to Southern Hemisphere (land only)					
AIS	1.12	1.21	0.95	1.03	1.17
EAIS	0.97	1.11	0.73	0.66	1.16
WAIS	1.36	1.40	1.34	1.58	1.18
AP	1.61	0.85	1.50	3.66	1.29

**Figure 3:**
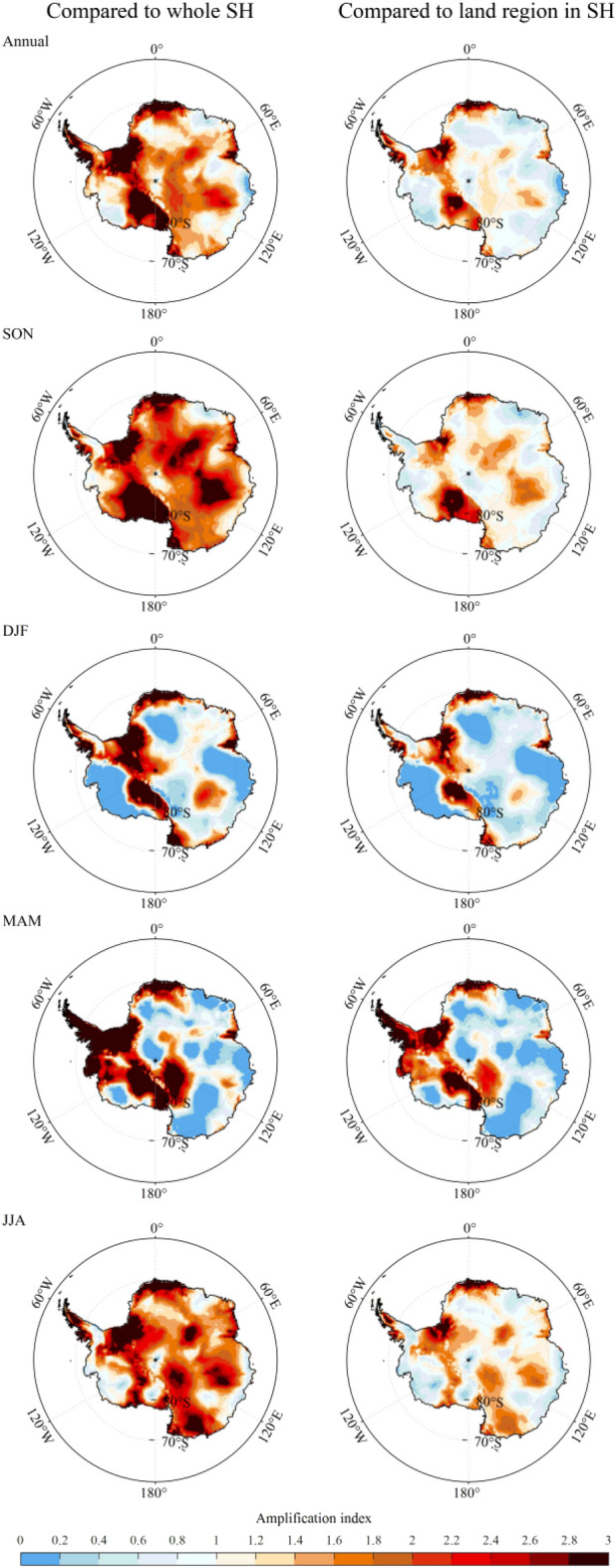
Spatial patterns of the annual and seasonal (spring, SON; summer, DJF; autumn, MAM; winter, JJA) amplification index over Antarctica. The map was drawn in the software MATLAB R2018b (https://ww2.mathworks.cn/products/matlab.html).

The spatial patterns of the annual and seasonal amplification index over the AIS are shown in Fig. 3, which reflects the regional differences of AnA more directly. Clearly, most parts of AIS experience warming amplification. Compared with the average temperature change in SH, strong annual AnA occurs in Ronne ice shelf and western side of AP, and this feature can also be observed in seasonal variations. The amplification signal dominates AIS in SON, and disappears in most areas of EAIS in summer and autumn. In winter, amplification domains EAIS, and disappears in Marie Byrd Land of WAIS. Compared with the average temperature change in land region of SH, AnA is obviously weakened in spring and winter, and strong spring amplification can be observed in Rose Ice Shelf.

### EDW and LDW characteristics in Antarctica during 1958–2020

**Figure 4:**
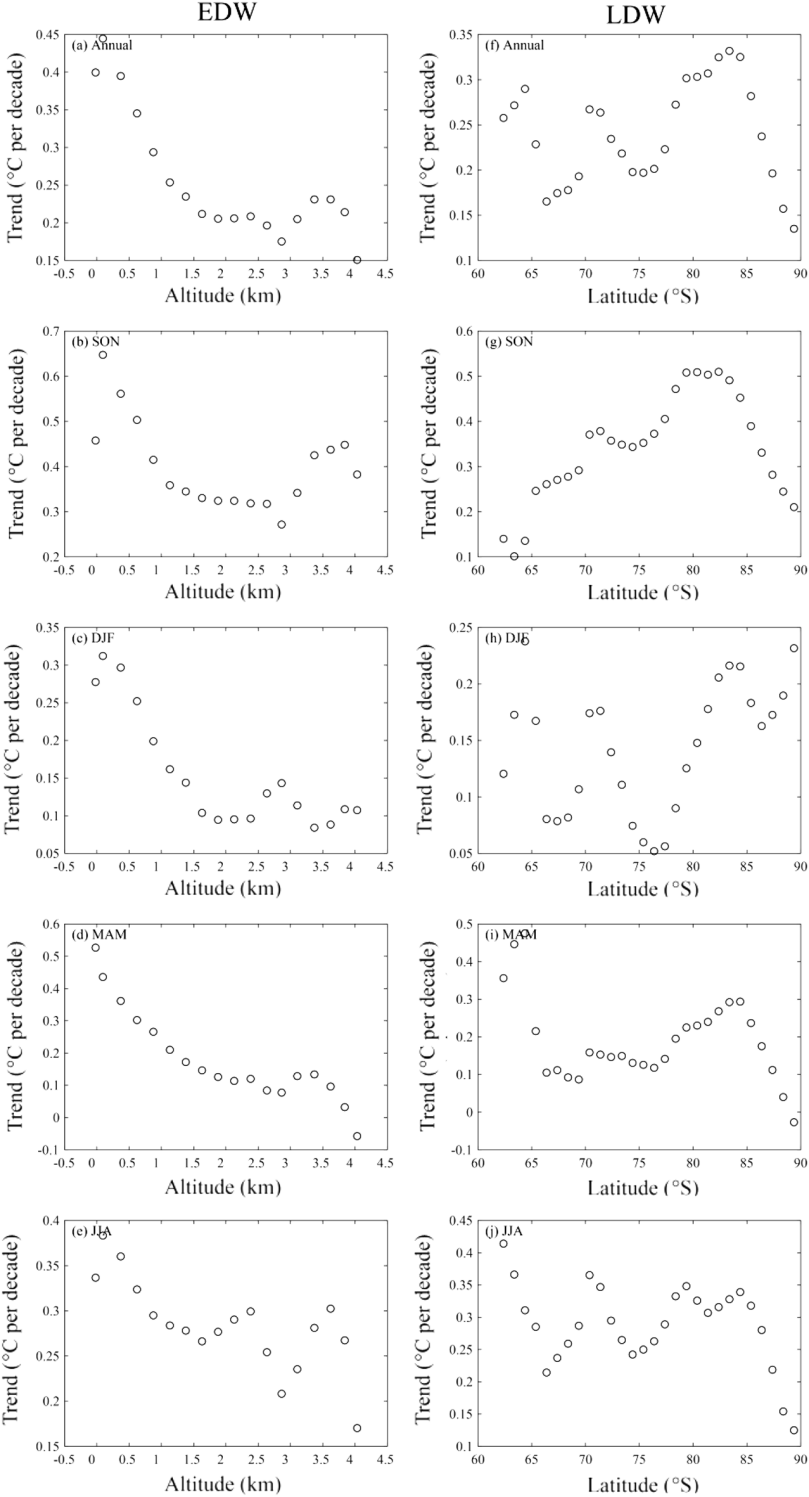
Annual and seasonal trends of EDW and LDW over Antarctica during the period 1958–2020 obtained from the elevation and latitude band methods, respectively. The mean temperature trends are plotted against 18 elevation ranks (**a**-**e**, starting at − 250 m in 250 m steps) and 28 latitude ranks (**f**–**j**, starting at 63 °S in 1.0°steps), respectively. The trends are all statistically significant at the 95% level.

Figure 4 illustrates the near-surface temperature trends as a function of altitude divided into 250 m bands and latitude divided into 1.0° bands. On annual scale, it is clear that the near-surface temperature trends decrease with elevation between 250 m and 3000 m, and it indicates the occurrence of negative EDW, with the altitudinal amplification trend of − 0.088 °C per decade^−1^ km^−1^ (*R*^2^ = 0.842, *p* < 0.001). In this altitude range, EDW exhibits seasonal difference, with most conspicuous negative EDW in austral autumn, and the altitudinal amplification trend is − 0.122 °C per decade^−1^ km^−1^ (*R*^2^ = 0.910, *p* < 0.001). In spring, the rate of near-surface warming decrease from 0.65 °C per decade at 0–250 m elevation band to 0.27 °C per decade at 2750–3000 m elevation band. In contrast, the near-surface warming increase with the altitude from 2500 m to 3000 m in summer, and from 1750 m to 2250 m in winter.

Clearly, the annual LDW can be observed, and the near-surface warming trends decrease with latitude between 70 °S and 75 °S, and the feature also appears between 83 °S and 90 °S, with the latitudinal amplification trend of − 0.016 °C per decade^−1^ degree^−1^ (*R*^2^ = 0.949, *p* < 0.001) and − 0.036 °C per decade^−1^ degree^−1^ (*R*^2^ = 0.983, *p* < 0.001), and the positive LDW between 75 °S and 83 °S is 0.018 °C per decade^−1^ degree^−1^ (*R*^2^ = 0.913, *p* < 0.001). In austral spring, autumn and winter, the negative LDW occurs between 83 °S and 90 °S, and the corresponding latitudinal amplification trends are − 0.049 °C per decade^−1^ degree^−1^ (*R*^2^ = 0.991, *p* < 0.001), − 0.057 °C per decade^−1^ degree^−1^ (*R*^2^ = 0.969, *p* < 0.001) and − 0.039 °C per decade^−1^ degree^−1^ (*R*^2^ = 0.916, *p* < 0.001), respectively, which indicates the strongest negative LDW in austral autumn. In DJF, the warming rates increase from 0.05 °C per decade at 76 °S to 0.22 °C per decade at 83 °S, with the amplification trend of 0.023 °C per decade^−1^ degree^−1^ (*R*^2^ = 0.953, *p* < 0.001).

On the whole, the results demonstrate that annual and seasonal warming over Antarctica is not only related to altitude, but also to latitude, and the strength of EDW is always higher than LDW.

### Physical mechanisms controlling EDW over the Antarctica

Snow/ice-albedo feedback, cloud feedback, atmospheric water vapor feedback and ozone change are thought to be the contributor of EDW in high mountain areas^3,5,60^. In AIS, most areas is mainly covered by ice and snow even in austral summer, and the albedo is extremely high, especially in the interior of the Antarctic inland^61^. Therefore, we only analyze the influence of near-surface specific humidity, surface downward long-wave radiation, surface downward short-wave radiation, total cloud cover, total column ozone and cloud base height in the EDW over AIS during the period 1958–2020.

**Figure 5:**
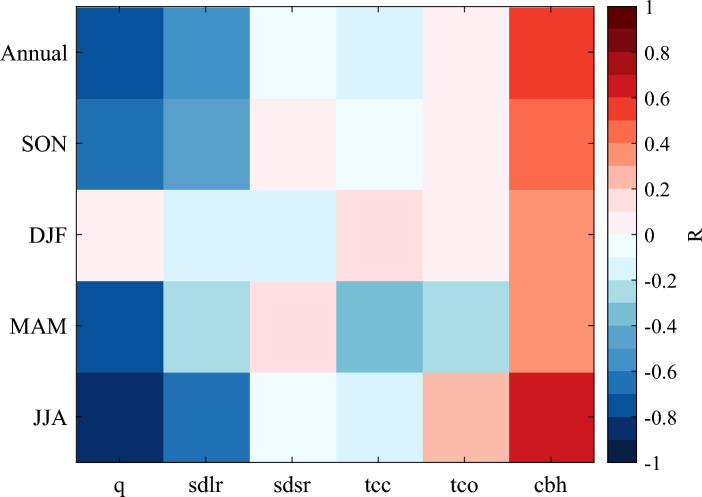
Annual and seasonal correlation coeffcient between the following variables—near-surface specific humidity (q), surface downward long-wave radiation (sdlr), surface downward short-wave radiation (sdsr), total cloud cover (tcc), total column ozone (tco), cloud base height (cbh)—and the elevation in Antarctica.

Figure 5 displays the annual and seasonal correlation coeffcients (R) between each of above factors and elevation. The annual R of specific humidity, surface downward long-wave radiation, surface downward short-wave radiation, total cloud cover, total column ozone, cloud base height and elevation are − 0.71, − 0.56, − 0.04, − 0.14, 0.08 and 0.57, respectively. On seasonal scale, surface downward long-wave radiation (cloud base height) always show negative (positive) correlation with elevation, particularly in winter, with R of − 0.61 (0.61). Except in summer, specific humidity is negatively correlated with elevation, and the most obvious correlation occurs in winter, and the correlation coefficient is − 0.84. This indicates that the specific humidity, surface downward long-wave radiation, total cloud cover and cloud base height may have an important impact on EDW in AIS.

**Figure 6:**
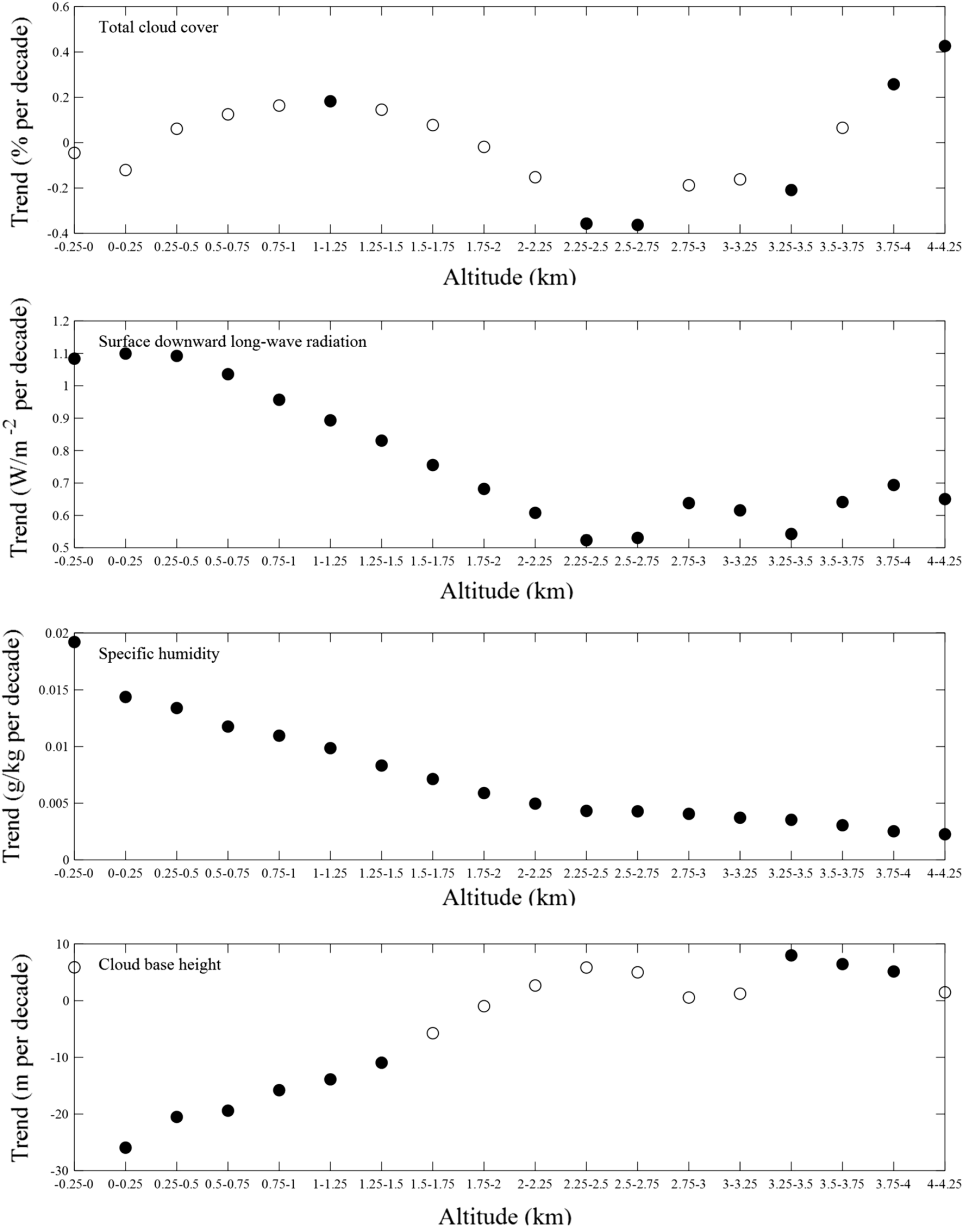
Linear trends of annual-mean total cloud cover, surface downward long-wave radiation, near-surface specific humidity and cloud base height for the period 1958–2020. Solid circles indicate that the trends are statistically significant at the 95% level.

On annual scale, the cloud cover in Antarctica decreases in the region band 1750–3750 m, and the value of downtrend varies linearly with latitude in the band 1750–2750 m (Fig. 6). In the band 0–2250 m, the magnitude of increasing trend in specific humidity decrease with increasing latitude, and the negative trend of cloud base height weakens with latitude and turns into an upward trend. The variations in surface downward long-wave radiation corresponds well to the change in specific humidity and cloud base height between 0 m and 2250 m (Fig. 5). The correlation coefficients between annual temperature tendency and variations in surface downward long-wave radiation is 0.84, and the corresponding value of total cloud cover, near-surface specific humidity and cloud base height is 0.12, 0.90 and − 0.58, respectively. The strong correlation also can be observed in austral spring and autumn. The correlation coefficients in SON between temperature trend and surface downward long-wave radiation is 0.86, and is 0.89 in MAM. Specific humidity and cloud base height also hows high correlation with the temperature trend, with the correlation coefficients of 0.91 and 0.66 in autumn, respectively. In addition, these factors except near-surface specific humidity have low ability to explain the winter EDW over AIS, and the correlation coefficients is lower than 0.20 in general.

Research have found that the increases in downward longwave radiation can explain more than 70% of the warming over AIS, which is related to the variations in atmospheric moisture loading and total column integrated cloud^62^. In altitude band, the high correlation coefficients between variations in surface downward long-wave radiation and near-surface specific humidity appears in autumn and winter, and the correlation coefficients are 0.89 and 0.97, respectively. In AIS, the change of surface downward long-wave radiation is positively correlated with total cloud cover particularly in austral summer, and it illustrates the negative correlation with cloud base height, and the correlation coefficient ranges from − 0.29 (autumn) to − 0.83 (spring).

Therefore, it can be deduced that the surface downward long-wave radiation is the key explanatory factor for EDW over AIS during the study period 1958–2020. It should be noted that the EDW may be influenced by several factors that have various feedbacks at the same time, and the mechanism needs to be further explored in the future research.

## Discussions

Both altitude and latitude affect the changes of Antarctic temperature. As a great concern, the similar phenomenon of EDW and LDW can also be captured in TP and Arctic^3,7,63^. EDW widely exists in high-elevation regions across the globe, however, this phenomenon does not always exist^64^. In our study, the EDW exists in AIS, and shows different characteristics at different altitude ranges. Moreover, the signal from altitude effect can be influenced by the noise from specific factors such as temperature inversion of low elevation stations^64,65^. In AIS, this effect in low altitude area might be very low, and probably masked as it accounts a very low percentage of the Antarctic terrain. In addition, the high altitude region concentrates on the East Antarctic inland, which is also in high latitude, and it may induce the coupling of EDW and LDW (such as the annual negative EDW in 3500–4000 m and annual negative LDW in 85–90 °S.

**Figure 7:**
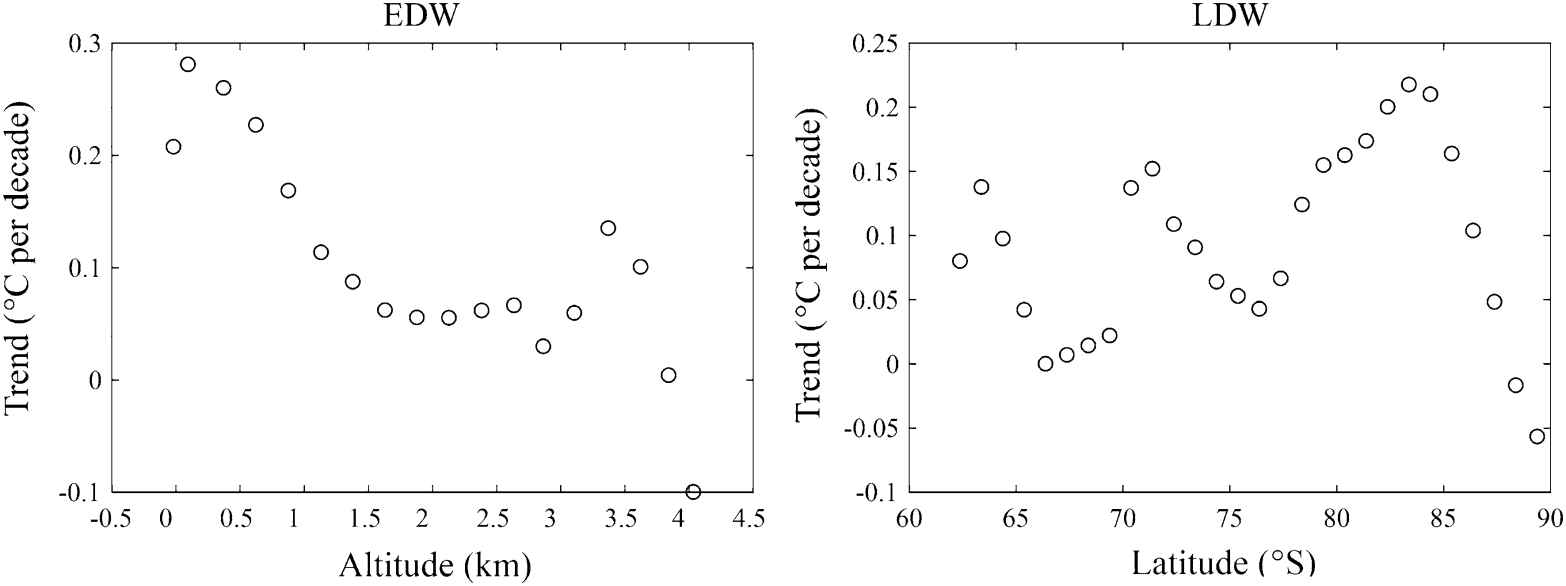
EDW and LDW trends in Antarctica during the period 1979–2020. The trends are all statistically significant at the 95% level.

This study depends on the reanalysis data ERA5, therefore, the results are inevitably affected by the accuracy of the data. In AIS, the observations data are scarce and the distribution of weather station is inhomogeneous^53^, which brings challenges to the performance of reanalysis data in representing Antarctic temperature. The reanalysis data has been assimilating satellite data since 1979, and it can reproduce the changes in Antarctic temperature. Compared to independent observations in AIS, ERA5 shows an outstanding performance, with correlation coefficients of 0.94 and mean bias of 0.08 °C on annual scale^53^, and it indicates that ERA5 shows low uncertainty in Antarctic temperature and the results from ERA5 are faithful. The ERA5 data extends back to 1950, which assimilate additional conventional observations and improve the use of early satellite data, and the quality of ERA5 in the period 1950–1978 has been significantly improved^66^. However, the observation record and satellite data are unavailable in AIS before 1957, and ERA5 may has an obvious deviation in retrieving Antarctic temperature before the International Geophysical Year. As shown in Fig. 7, for the period 1979–2020, the annual negative EDW can be observed in 250–2500 m, with the altitudinal amplification trend is − 0.096 °C per decade^−1^ km^−1^ (*R*^2^ = 0.836, *p* < 0.001), and the altitude higher than 3500 m also shows negative EDW. On annual scale, negative LDW appears between 70 °S to 75 °S and 83 °S to 90 °S, and positive LDW occurs in the area between the two latitude bands. Overall, the EDW and LDW in 1979–2020 is basically consistent with that in 1958–2020, although there are slight differences in the magnitude of altitudinal amplification trend and latitudinal amplification trend.

From the above results obtained from the elevation band method and latitude band method, the EDW is stronger compared with the LDW over AIS. Many researches have explored the physical mechanisms that can lead to EDW, and TP is a hot research topic for studying the altitude amplification effect. Seasonal snow cover varies with altitude over the TP, and snow/ice-albedo feedback can influence EDW by changing the surface-absorbed solar radiation^13,14^. Differently, the impact of snow cover on Antarctic EDW is almost negligible. Cloud can influence the short-wave and long-wave radiation, thereby further affecting the EDW^3,67,68^. Similarly, the long-wave radiation is critical to the EDW over AIS. However, deeper research should be conducted to explore the possible physical mechanism responsible for the EDW and LDW, and assess the future changes of EDW and LDW and their broader consequences in AIS.

## Conclusions

Based on the mean temperature series (1958–2020) of ERA5 reanalysis data, this study explores whether there are EDW and LDW in the Antarctic warming based on elevation and latitude band methods. The results show that latitude and altitude jointly contribute to Antarctic warming, and it shows different characteristics in different ranges. The negative EDW can be detected between 250 m and 2500 m except in austral winter, and the magnitude of altitudinal amplification trend is strongest in austral autumn. The negative LDW appears between 83 °S and 90 °S except in summer, and is also most conspicuous in autumn. Annual and summer positive LDW can be observed between 75 °S and 83 °S. In addition, the magnitude of altitudinal amplification trends are higher than latitudinal amplification trends.

This study quantifies the contribution of altitude and latitude to the Antarctic amplification, and confirms that the surface downward long-wave radiation is one of the most important factor for explaining the EDW in Antarctica, which is also influenced by the specific humidity, total cloud cover and cloud base height.

Finally, due to the harsh environment in Antarctica, the long-term observational datasets are scarce and the weather stations are very rare. The input data of observations can affect the accuracy of reanalysis, and then inevitably affect the results. Therefore, further research is also required to implement the comparative studies with different types of data sources and with longer time series and more high-quality observations over Antarctica.

## Data Availability

The monthly climate data of Fifth Generation Global Atmospheric reanalysis (ERA5) used in this study are openly available at https://cds.climate.copernicus.eu/#!/search?text=ERA5&type=dataset.
